# Assessing vaccination priorities for different ages and age-specific vaccination strategies of COVID-19 using an SEIR modelling approach

**DOI:** 10.1371/journal.pone.0261236

**Published:** 2021-12-22

**Authors:** Cong Yang, Yali Yang, Yang Li

**Affiliations:** 1 Fundamentals Department, Air Force Engineering University, Xi’an, Shaanxi, China; 2 Command and Control Center of Wenchang Spacecraft Launch Site, Wenchang, Hainan, China; Konkuk University, KOREA, REPUBLIC OF

## Abstract

In the past year, the global epidemic situation is still not optimistic, showing a trend of continuous expansion. With the research and application of vaccines, there is an urgent need to develop some optimal vaccination strategies. How to make a reasonable vaccination strategy to determine the priority of vaccination under the limited vaccine resources to control the epidemic and reduce human casualties? We build a dynamic model with vaccination which is extended the classical SEIR model. By fitting the epidemic data of three countries—China, Brazil, Indonesia, we have evaluated age-specific vaccination strategy for the number of infections and deaths. Furthermore, we have evaluated the impact of age-specific vaccination strategies on the number of the basic reproduction number. At last, we also have evaluated the different age structure of the vaccination priority. It shows that giving priority to vaccination of young people can control the number of infections, while giving priority to vaccination of the elderly can greatly reduce the number of deaths in most cases. Furthermore, we have found that young people should be mainly vaccinated to reduce the number of infections. When the emphasis is on reducing the number of deaths, it is important to focus vaccination on the elderly. Simulations suggest that appropriate age-specific vaccination strategies can effectively control the epidemic, both in terms of the number of infections and deaths.

## 1 Introduction

At the beginning of 2020, there has been a global outbreak of COVID-19, which had a significant impact on people’s daily lives [1, 2]. In the past year, every country is actively responding to the severe challenge brought by COVID-19 and all pay great efforts for it. At present social distancing along with previously known traditional medicines can act as quick and short-term alternatives for treating this viral flu [3]. In the absence of any specific antiviral vaccine, various non-pharmacological measures coupled with lockdown have been employed to combat this infection [4]. Unfortunately, there are still many countries where the epidemic is spreading, so research and use of the vaccine is an important way to beat with COVID-19.

There are about 100 COVID-19 vaccines in research in the past year, many of which have made significant progress. Some vaccines have already been approved for use, and a large number are undergoing phase 3 clinical trials [5]. However, the production efficiency of the vaccine and the storage requirements of the vaccine mean that the amount of vaccine at this stage cannot reach the number of individuals who need vaccination. Therefore, how to develop vaccination strategies based on priority guidelines is currently a major concern in many countries. In the early stages of the COVID-19 outbreak, research focused on assessing the impact of the size of the epidemic, the number of deaths, and the base number of relapses on the development of COVID-19 [6–8]. With the global outbreak of the epidemic, research focus has rapidly shifted to the formulation and evaluation of the effectiveness of measures [9–12]. With the advent of vaccination, it is becoming increasingly popular to develop vaccination strategies to maximize the impact of vaccines in controlling the epidemic [13–17].

Application of mathematical models to disease surveillance data can be used to address both scientific hypotheses and disease-control policy questions [18]. There have been several modelling studies [19–23] in which researchers have tried to identify the best control strategies for vaccination in the prevention and control of epidemics. Considering the large limitation of COVID-19 vaccine supply, the article used an age-specific SEIR model to study five different priority vaccination strategies in the United States. Many studies have proved that age is an important factor affecting the susceptibility to infectious diseases and mortality [24–28]. So, it’s important to take age structure into account when considering vaccination strategies. Different countries have different age structures and different age-structure groups have different networks of connections [29]. Bassey B E and Atsu J U analyzed the global stability of COVID-19 in multi-therapeutic and non-pharmacological treatment protocols [30]. On this basis, we extend an age structure SEIR model to determine the optimal age specific vaccination distribution with different age structures in different countries.

The main purpose of this study is to use a mathematical model to quantitatively analyze how to design the optimal vaccination strategy under the number of vaccines are limited. The other sections of this paper are arranged as follows: In section 2, we mainly introduce the COVID-19 propagation dynamics model with vaccination. In section 3, the validity of the model was verified, and the unknown parameters are estimated by fitting COVID-19 epidemic data. In section 4, we mainly propose the vaccination strategy with age structure. In section 5, the suitability of vaccination strategies is simulated by using different values of the parameters such as *R*_0_, *I*_*C*_ and *D*_*C*_ to assess age-structure characteristics. We make the conclusions and give suggestions in section 6.

## 2 An SEIR model with vaccine chamber

**Fig 1:**
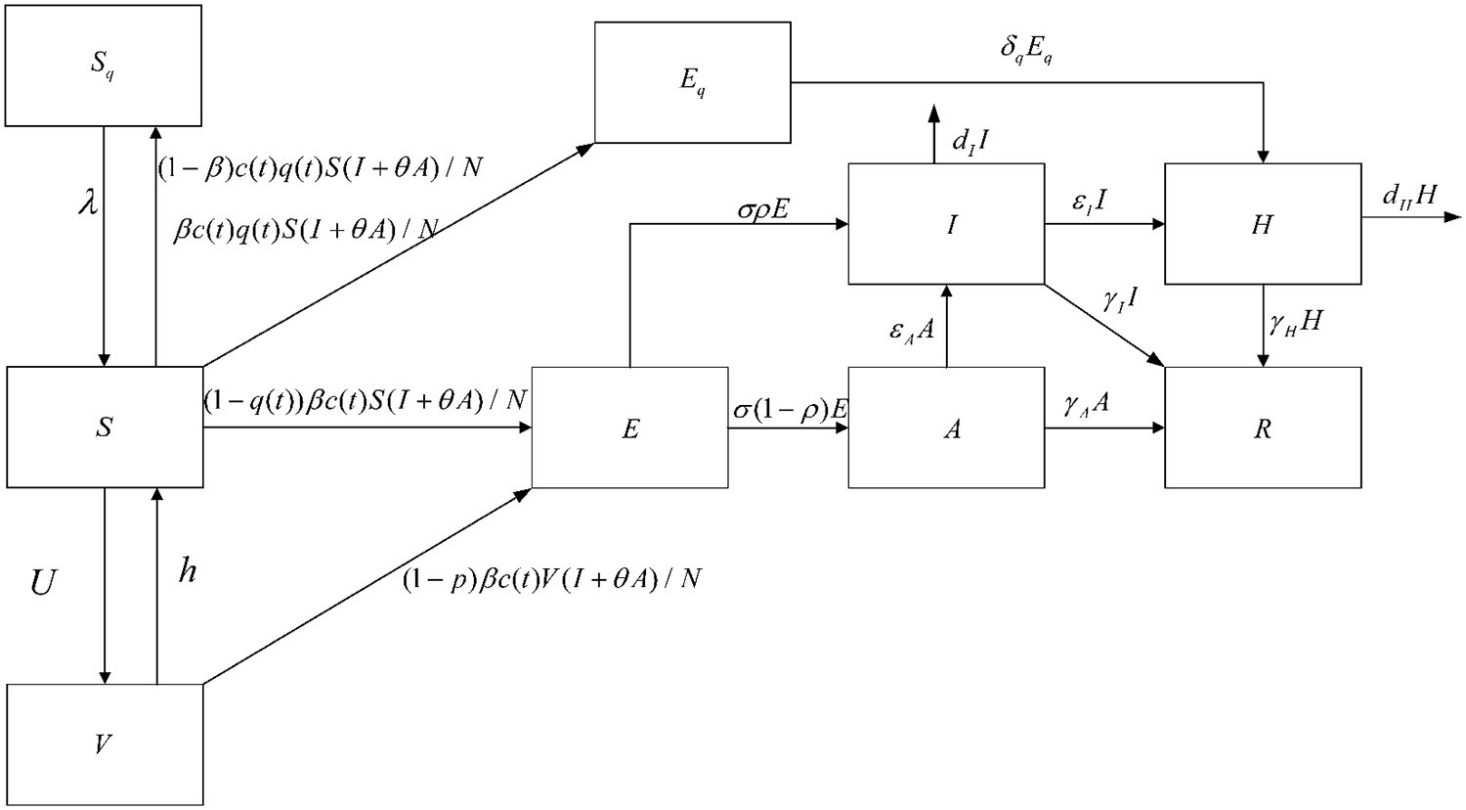
The transmission diagram for model (2.1).

With the continuous development of the epidemic, the modelling of COVID-19 has been extensively studied. Vaccination is a key link in the prevention and transmission of infectious diseases. Based on previous mathematical modelling, we developed a new model of COVID-19 transmission with a focus on vaccination. The population was divided into nine compartments: the susceptible (*S*), the quarantined susceptible (*S*_*q*_), the isolated exposed (*E*_*q*_), the vaccine (*V*), the exposed (*E*), infectious with symptoms (*I*), asymptomatic infectious(*A*), the hospitalized (*H*)and recovered (*R*). Because of the government’s quarantine measures, the ratio of *q* individuals who contact with the infected will be quarantined. The quarantined population is divided into two compartments, *E*_*q*_ or *S*_*q*_, depending on whether they are effectively infected or not. The other individuals exposed to the virus in the 1 − *q* portion are not tracked and therefore transferred to the *E* once they are effectively infected, or remain in the susceptible compartment *S*. The parameters with their definitions are presented in Table 1. The transmission diagram for model (2.1) is shown in Fig 1. The dynamics model is given by:

$$\begin{array}{c} \left\{ \begin{array}{c} \frac{dS}{dt}=-c\left(t\right)\beta S\left(I+\theta A\right)/N-\left(1-\beta \right)c\left(t\right)q\left(t\right)S\left(I+\theta A\right)/N-U+hV+\lambda S_{q},\\ \frac{dS_{q}}{dt}=\left(1-\beta \right)c\left(t\right)q\left(t\right)S\left(I+\theta A\right)/N-\lambda S_{q},\\ \frac{dE_{q}}{dt}=\beta c\left(t\right)q\left(t\right)S\left(I+\theta A\right)/N-\delta_{q}E_{q},\\ \frac{dV}{dt}=U-hV-\beta c\left(t\right)\left(1-p\right)V\left(I+\theta A\right),\\ \frac{dE}{dt}=\left(1-q\left(t\right)\right)\beta c\left(t\right)S\left(I+\theta A\right)/N+\left(1-p\right)\beta c\left(t\right)V\left(I+\theta A\right)/N-\sigma E,\\ \frac{dI}{dt}=\sigma \rho E+\varepsilon_{A}A-\left(\varepsilon_{I}\left(t\right)+d_{I}+\gamma_{I}\right)I,\\ \frac{dA}{dt}=\sigma \left(1-\rho \right)E-\gamma_{A}A-\varepsilon_{A}A,\\ \frac{dH}{dt}=\varepsilon_{I}\left(t\right)I+\delta_{q}E_{q}-\left(d_{H}+\gamma_{H}\right)H,\\ \frac{dR}{dt}=\gamma_{1}I+\gamma_{A}A+\gamma_{H}H. \end{array}\right. \end{array}$$ (1)

Where *N* = *S* + *S*_*q*_ + *E*_*q*_ + *V* + *E* + *I* + *A* + *H* + *R* is the total population.

**Table 1:** Compartment definitions.

Compartment	Definition
*S*	Susceptible population
*S*_*q*_	Quarantined susceptible population
*E*_*q*_	Quarantined exposed population
*V*	Vaccinated population
*E*	Exposed population
*I*	Infected symptomatic population
*A*	Infected symptomatic population
*H*	Confirmed and hospitalized population
*R*	Recovered population
*D*	Death population

Considering that with the continuous development of the epidemic, the control measures of local governments have been continuously improved, and the isolation rate of people exposed to the virus has been continuously increased. After the effective understanding of COVID-19, the diagnostic rate for patients has also increased with time. The isolation rate and diagnosis rate increased with time in the following relationship [7, 31].

$$\begin{array}{c} q\left(t\right)=\left(q_{0}-q_{m}\right)\text{exp}\left(-r_{q}t\right)+q_{m}. \end{array}$$

$$\begin{array}{c} \varepsilon_{I}\left(t\right)=\left(\varepsilon_{I0}-\varepsilon_{Ib}\right)\text{exp}\left(-r_{\varepsilon }t\right)+\varepsilon_{Ib}. \end{array}$$

For endemic region, we hypothesize that exposure rates will decrease over time as people pay more and more attention to masks and socialize more and more reasonably.

$$\begin{array}{c} c\left(t\right)=\left(c_{0}-c_{m}\right)\text{exp}\left(-r_{c}t\right)+c_{m}. \end{array}$$

The basic reproduction number for model (2.1) can be calculated according to [32]:

$$\begin{array}{c} R_{0}=\frac{\left(\rho \gamma_{A}+\varepsilon_{A}\right)}{\left(\varepsilon_{I}+d_{I}+\gamma_{I}\right)\left(\gamma_{A}+\varepsilon_{A}\right)N}\left(\left(1-q\right)\beta cS_{0}+\left(1-p\right)\beta V_{0}\right)+\frac{\theta (1-\rho )}{(\gamma_{A}+\varepsilon_{A})N}\left(\left(1-q\right)\beta cS_{0}+\left(1-p\right)\beta V_{0}\right). \end{array}$$

Where *S*_0_ is the initial value of the susceptible individuals, *V*_0_ is the initial value of the vaccine.

## 3 Data and parameter calibration

**Fig 2:**
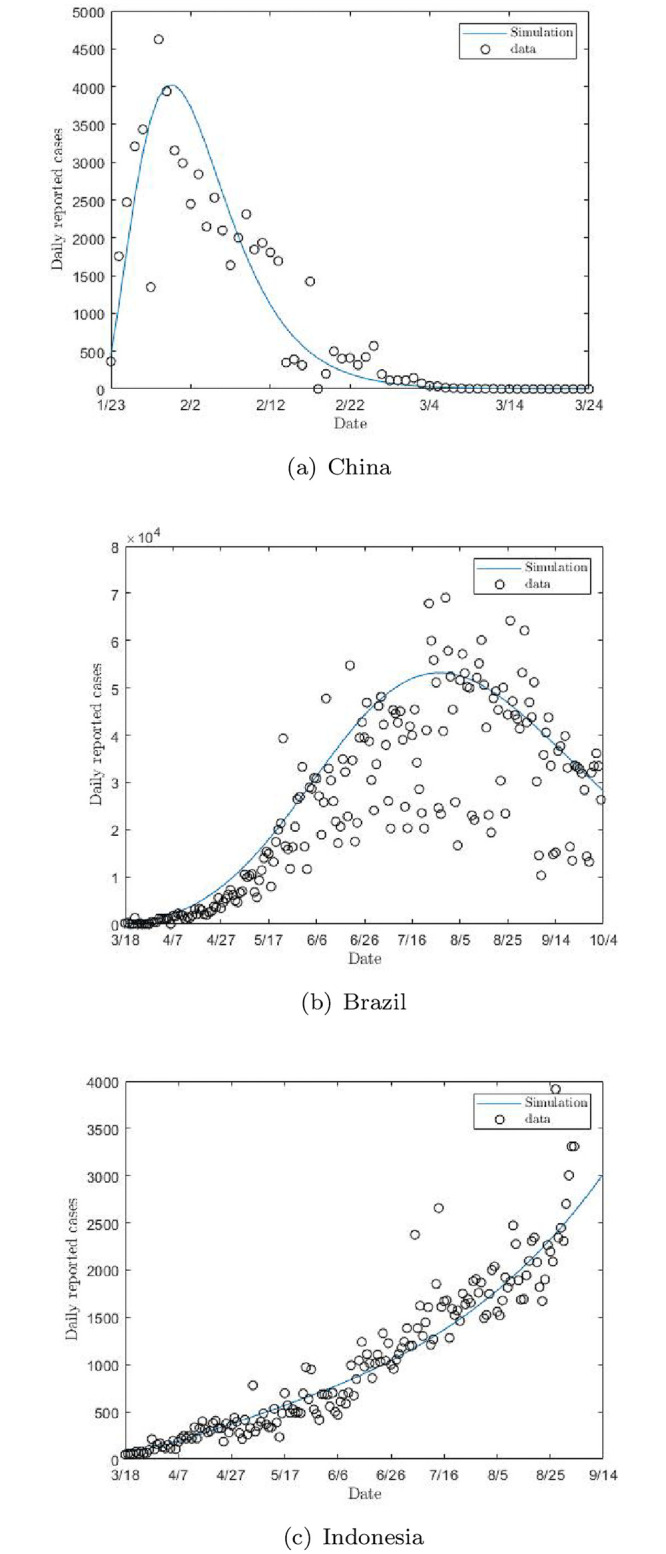
Observed daily new cases (dots) and model fitting results (solid curve) for China (a), Brazil (b) and Indonesia (c).

In order to accurately estimate the parameters of the model, some parameters are determined from existing studies or data. The data for the population age distribution are obtained from https://www.populationpyramid.net/japan/2019/ [33]. The data of daily reported COVID-19 cases were obtained from https://github.com/CSSEGIS and Data [34]. We fit the new confirmed cases per day in each country to retrieve the parameters of the model with historical data. Using the parameters obtained from the inversion, we plot the fitting results for three countries. The transmission probability from A or I to S per contact (*β*) is different in different area. The infection ability of symptomatic infected individuals is weaker than that of symptomatic infected individuals and *θ* is a correction factor which is assumed to be 0.0232 [6]. The asymptomatic infected individuals could become symptomatic infected individuals and *ε*_*A*_ is assumed to be 0.4. Vaccination does not represent permanent immunity. Over time, a certain percentage of people lose vaccine protection, a ratio of *h* which is assumed to 0.006. Vaccination protection rate cannot reach 100%, so the proportion of vaccine protection rate *p* is assumed to be 0.65. Transition rate of exposed individuals to the infected *σ* is assumed to be 1/5.2 [35]. With the expiration of isolation and other reasons, individuals who are released from isolation will return to the susceptible group, that is λ = 1/14 [6]. Compartment values used in the simulations show in Table 2. Estimates of case fatality rates may vary slightly from country to country due to differences in prevention, control and mitigation policies implemented, as well as the availability and availability of health care [36]. Early studies [37, 38] have shown that delaying the detection of infected cases not only increases the probability of spreading the virus to others (most likely family members, colleagues, and friends) but also makes the infection worse in some cases, thereby increasing the case fatality ratio [39]. During the epidemic in Wuhan, more than 42,000 medical personnel from all over the country were sent to Hubei, more than 35,000 of them were in Wuhan, which doubled the medical personnel manpower in Wuhan. In addition, the hospital of Thunder Mountain and Fire Shenshan is vital to the treatment of patients, but it is impossible to quantify [40]. The values of parameters related to COVID-19 and its transmission are obtained from the relevant literature, and other parameters are obtained by data fitting (Tables 3 and 4). According to the estimated value, the daily new cases in the three regions can be fitted and compared with the actual data, as shown in Fig 2.

**Table 2:** Compartment values used in the simulations.

Compartment	China	Brazil	Indonesia	Source
*S*_0_	2.17 × 10^7^	209598000	270625600	[41]
*S*_*q*0_	7347	1522	1905	[41]
*E*_*q*0_	60	639	612	Estimated
*V*_0_	0	0	0	Estimated
*E*_0_	29794	1478	1012	[41]
*I*_0_	3413	1147	174	Estimated
*A*_0_	4820	800	159	Estimated
*H*_0_	771	367	197	[34]
*R*_0_	34	2	11	[34]

**Table 3:** Definition of parameters.

Parameter	Definition
*β*	Transmission probability from *A* or *I* to *S* per contact
*c*_0_	Contact rate at the initial time
*c*_*m*_	Minimum contact rate with control
*r*_*c*_	Exponential decreasing rate of contact rate
*θ*	Correction factor for transmission probability of asymptomatic infectious
*q*_0_	Quarantined rate at the initial time
*q*_*m*_	Maximum quarantined rate with control
*r*_*q*_	Exponential increasing rate of quarantined rate
λ	Releasing rate of quarantined susceptible
*U*	Number of individuals vaccinated
*h*	The rate of vaccine failure
*σ*	Transition rate of exposed individuals to the infectious (*A* or *I*) class
*δ*_*q*_	Diagnose rate of quarantined individuals
*p*	Probability of vaccine protection
*ρ*	Ratio of symptomatic infection
*ε*_*A*_	The probability from *A* to *I*
*ε*_*I*0_	Diagnose rate of infected individuals at the initial time
*ε*_*Ib*_	Maximum diagnose rate of infected individuals
*r*_*ε*_	Exponential increasing rate of diagnose rate
*d*_*I*_	Disease-induced death rate of infected individuals
*d*_*H*_	Disease-induced death rate of hospitalized individuals
*γ*_*I*_	Recovery rate of infected individuals
*γ*_*A*_	Recovery rate of asymptotic infectious individuals
*γ*_*H*_	Recovery rate of hospitalized individuals

**Table 4:** Parameters used in the simulations.

Parameter	China	Brazil	Indonesia	Sources
*β*	0.06	0.13	0.06	Estimated
*c*_0_	14.76	12.0	12.19	[42]
*c*_*m*_	3	6.71	6	[42]
*r*_*c*_	1.01	0.08	0.05	Estimated
*θ*	0.0232	0.0232	0.0232	Estimated
*q*_0_	0.00001	0.001	0.01	[42]
*q*_*m*_	0.95	0.49	0.12	[42]
*r*_*q*_	0.08	0.01	0.28	[42]
λ	1/14	1/14	1/14	[6]
*U*	-	-	-	According to scenario
*h*	-	-	-	According to scenario
*σ*	1/5.2	1/5.2	1/5.2	[35]
*δ*_*q*_	0.35	0.12	0.10	Estimated
*p*	0.33	0.33	0.33	[42]
*ρ*	0.9	0.6	0.54	[6]
*ε*_*A*_	1/5	1/5	1/5	Estimated
*ε*_*I*0_	0.05	0.10	0.05	Estimated
*ε*_*Ib*_	0.6	0.2	0.11	Estimated
*r*_*ε*_	0.05	0.19	0.5	Estimated
*d*_*I*_	0.01	0.004	0.01	Estimated
*d*_*H*_	0.2	0.2	0.2	[22]
*γ*_*I*_	0.07	0.14	0.05	Estimated
*γ*_*A*_	0.15	0.14	0.06	[19]
*γ*_*H*_	0.12	0.14	0.14	Estimated

## 4 Methods

### 4.1 Model

In daily life, the prevalence of infectious diseases is affected by different levels of population heterogeneity. Age differences among individuals in a group can lead to differences in activity levels, infectivity, healing ability and susceptibility. Therefore, it is necessary to take age structure into account when considering vaccination models. Global stability of equilibrium and uniqueness of existence are the most important theories.

$$\begin{array}{c} \left\{ \begin{array}{c} \frac{dS^{\left(l\right)}\left(t\right)}{dt}=-S^{\left(l\right)}\left(t\right)(\sum_{m=1}^{K}\frac{c_{lm}\beta_{l}}{N}\left(I^{\left(m\right)}\left(t\right)+\theta A^{\left(m\right)}\left(t\right)\right)+\sum_{m=1}^{K}\frac{c_{lm}q\left(1-\beta_{l}\right)}{N}\left(I^{\left(m\right)}\left(t\right)+\theta A^{\left(m\right)}\left(t\right)\right))+\\ \;\;\;\lambda {S_{q}}^{\left(l\right)}\left(t\right)-U^{\left(l\right)}+hV^{\left(l\right)}\left(t\right),\\ \frac{dS_{q}^{\left(l\right)}\left(t\right)}{dt}=S^{\left(l\right)}\left(t\right)(\sum_{m=1}^{K}\frac{\left(1-\beta_{l}\right)c_{lm}q}{N}\left(I^{\left(l\right)}\left(t\right)+\theta A^{\left(l\right)}\left(t\right)\right))-\lambda S_{q}^{\left(l\right)}\left(t\right),\\ \frac{dE_{q}^{\left(l\right)}\left(t\right)}{dt}=S^{\left(l\right)}\left(t\right)(\sum_{m=1}^{K}\frac{\beta_{l}c_{lm}q}{N}\left(I^{\left(l\right)}\left(t\right)+\theta A^{\left(l\right)}\left(t\right)\right))-\delta_{q}{E^{\left(l\right)}}_{q}\left(t\right),\\ \frac{dV^{\left(l\right)}\left(t\right)}{dt}=U^{\left(l\right)}-hV^{\left(l\right)}\left(t\right)-\sum_{m=1}^{K}\frac{c_{lm}\beta_{l}\left(1-p\right)}{N}V^{\left(l\right)}\left(t\right)\left(I^{\left(l\right)}\left(t\right)+\theta A^{\left(l\right)}\left(t\right)\right),\\ \frac{dE^{\left(l\right)}\left(t\right)}{dt}=S^{\left(l\right)}\left(t\right)\sum_{m=1}^{K}\frac{c_{lm}\beta_{l}}{N}\left(1-q\right)\left(I^{\left(m\right)}\left(t\right)+\theta A^{\left(m\right)}\left(t\right)\right)-\sigma E^{\left(l\right)}\left(t\right)\\ \;\;\;+\sum_{m=1}^{K}\frac{c_{lm}\beta_{l}\left(1-p\right)}{N}V^{\left(l\right)}\left(t\right)\left(I^{\left(l\right)}\left(t\right)+\theta A^{\left(l\right)}\left(t\right)\right)/N,\\ \frac{dI^{\left(l\right)}\left(t\right)}{dt}=\sigma \rho E^{\left(l\right)}\left(t\right)+\varepsilon_{A}A^{\left(l\right)}\left(t\right)-\left(\varepsilon_{I}+d_{I}+\gamma_{1}\right)I^{\left(l\right)}\left(t\right),\\ \frac{dA^{\left(l\right)}\left(t\right)}{dt}=\sigma \left(1-\rho \right)E^{\left(l\right)}\left(t\right)-\gamma_{A}A^{\left(l\right)}\left(t\right)-\varepsilon_{A}A^{\left(l\right)}\left(t\right),\\ \frac{dH^{\left(l\right)}\left(t\right)}{dt}=\varepsilon_{I}I^{\left(l\right)}\left(t\right)+\delta_{q}{E^{\left(l\right)}}_{q}\left(t\right)-\left(d_{H}+\gamma_{H}\right)H^{\left(l\right)}\left(t\right),\\ \frac{dR^{\left(l\right)}\left(t\right)}{dt}=\gamma_{1}I^{\left(l\right)}\left(t\right)+\gamma_{A}A^{\left(l\right)}\left(t\right)+\gamma_{H}H^{\left(l\right)}\left(t\right), \end{array}\right. \end{array}$$ (2)

Where *l*, *K* = 1, 2, 3, 4.

In order to consider the effectiveness of vaccination strategies at different ages in different countries (such as China, Brazil and Indonesia), we divided the entire population into four age groups (0-4), (5-19), (20-64) and (65-) based according to the contact data [31]. *c*_*lm*_ is the contact rate between a susceptible individual in the *l*−*th* group and an infected individual in the *m*−*th* group and *β*_*l*_ is the probability of infected individuals transmission per contact in the *l*−*th* group.

To compare the effectiveness of different vaccination strategies in controlling the outbreak, we considered two vaccination strategies: the first is a uniform vaccination strategy, where the same number of people in each age group are vaccinated at the same time. Another is the age structure of vaccination strategies. In the case of the uniform vaccination strategy, *U*^(1)^ = *U*^(2)^ = *U*^(3)^ = *U*^(4)^. In the age structured vaccination strategy, in order to keep the total number of people vaccinated per day the same, we assumed $\sum_{i=1}^{4}U^{\left(i\right)}=K$, which *K* is a fixed numerical. Considering that Beta distribution is defined in a finite interval and its density function is very flexible (it can be either unimodal or *U*-shaped). In addition, the uniform distribution is a special case of the beta distribution.

The basic reproduction number can be calculated according to [32], which is the principal eigenvalue of following matrix Λ:

$$\begin{array}{c} \begin{array}{cc} \Lambda = & \frac{\left(\rho \gamma_{A}+\varepsilon_{A}\right)\left(1-q\right)}{N\left(\varepsilon_{I}+d_{I}+\gamma_{I}\right)\left(\gamma_{A}+\varepsilon_{A}\right)}[\begin{array}{cccc} \beta_{11}c_{1}S_{10} & \beta_{12}c_{1}S_{10} & \beta_{13}c_{1}S_{10} & \beta_{14}c_{1}S_{10}\\ \beta_{21}c_{2}S_{20} & \beta_{22}c_{2}S_{20} & \beta_{23}c_{2}S_{20} & \beta_{24}c_{2}S_{20}\\ \beta_{31}c_{3}S_{30} & \beta_{32}c_{3}S_{30} & \beta_{33}c_{3}S_{30} & \beta_{34}c_{3}S_{30}\\ \beta_{41}c_{4}S_{40} & \beta_{42}c_{4}S_{40} & \beta_{43}c_{4}S_{40} & \beta_{44}c_{4}S_{40} \end{array}]\\ & +\frac{\left(1-\rho )\theta (1-q\right)}{N(\gamma_{A}+\varepsilon_{A})}[\begin{array}{cccc} \beta_{11}c_{1}S_{10} & \beta_{12}c_{1}S_{10} & \beta_{13}c_{1}S_{10} & \beta_{14}c_{1}S_{10}\\ \beta_{21}c_{2}S_{20} & \beta_{22}c_{2}S_{20} & \beta_{23}c_{2}S_{20} & \beta_{24}c_{2}S_{20}\\ \beta_{31}c_{3}S_{30} & \beta_{32}c_{3}S_{30} & \beta_{33}c_{3}S_{30} & \beta_{34}c_{3}S_{30}\\ \beta_{41}c_{4}S_{40} & \beta_{42}c_{4}S_{40} & \beta_{43}c_{4}S_{40} & \beta_{44}c_{4}S_{40} \end{array}] \end{array} \end{array}$$ (3)

$$\begin{array}{c} R_{0}=g\left(\Lambda \right), \end{array}$$

Where *g*(⋅) denotes the spectral radius of a matrix.

### 4.2 Evaluation of the optimal age-specific vaccination distribution

The basic reproduction number *R*_0_, the cumulative number of infections *I*_*C*_ and the cumulative number of deaths *D*_*C*_ are the key indicators to evaluate the severity of infectious diseases and the public health situation, so we use it in this study to evaluate the effectiveness of different vaccination strategies. To reduce the number of parameters, the beta distribution of age-specific vaccine strategies can be uniquely determined by parameters *α* and *β*, which we have evaluated as a function of two parameters, which we can evaluate the three endpoints by converting them into functions. We assume that the vaccine is administered at a fixed rate for 180 days. The optimal age-specific vaccination distribution is obtained by minimizing the three endpoints from vaccine initiation time *T* to *T*+ 180 days to obtain optimal *α* and *β*. For different countries, we assume that the timing of vaccination is different. Considering that the epidemic in China has been well controlled by now and the sporadic outbreaks in different cities are mainly due to overseas imports, we assume that all people in China are susceptible and one infected individual is imported from overseas to potentially initiate a new epidemic. We use the interior point method to optimize the three endpoint functions. Then, according to different actual needs, the optimal age-specific vaccination distribution in accordance with the actual situation of each country are determined. In practical application, we suggest using *Matlab* embedded program ODE to solve the new dynamics system.

## 5 Results

### 5.1 The optimal age-specific vaccination distribution

**Fig 3:**
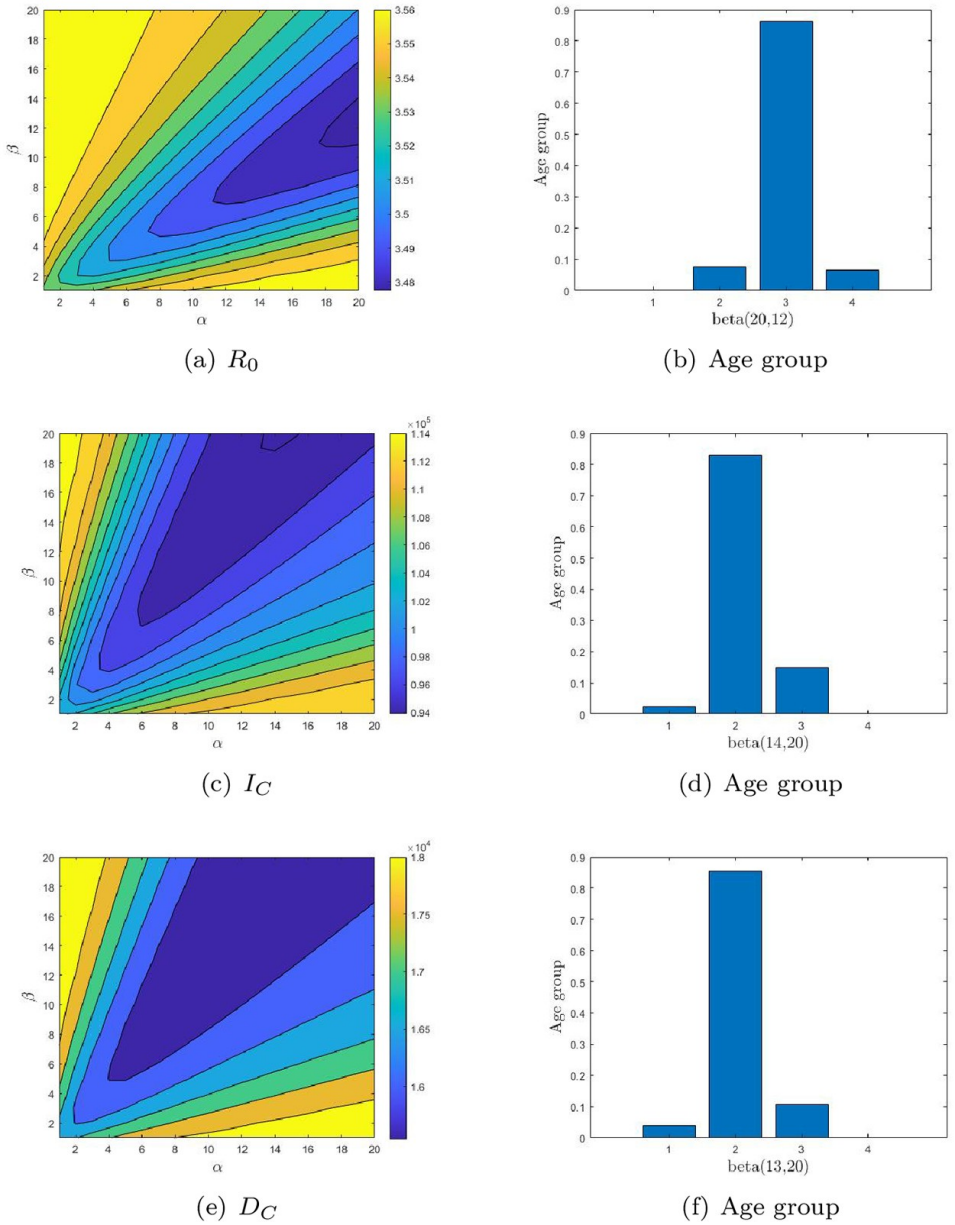
The contour plot of the three endpoints: The basic reproduction number (*R*_0_, 1st row), the cumulative number of infections (*I*_*C*_, 2nd row) and the cumulative number of deaths (*D*_*C*_, 3rd row) for China. The optimal age-specific vaccination distributions for these three endpoints are shown in (b), (d) and (f) respectively.

We evaluate the impact of vaccine allocation strategies with different age structures on COVID-19 outbreaks and determined the optimal age-specific vaccination distribution (OAVD) for different countries by minimizing three endpoints. We added unvaccinated cases as baseline and for comparative analysis under various vaccination strategies. Vaccine coverage rates vary from country to country, so we determined the vaccination rate to be 0.1% after comprehensive consideration. Fig 3 shows a contour plot of the three endpoints of the Chinese OAVDs and functions.

In all three countries, both *I*_*C*_ and *D*_*C*_ were significantly reduced under the vaccination strategy compared with no vaccination (See Table 5). And the difference is different between vaccine strategies compared to no vaccination. It can be seen that to minimize *R*_0_, people between the ages of 5 and 19 should be given priority for China. There are significant differences between the endpoints of *R*_0_ and those of *I*_*C*_ and *D*_*C*_ by OAVDs. The OAVD obtained by minimized *I*_*C*_ and *D*_*C*_ is similar (See Fig 3). If the minimum *I*_*C*_ and *D*_*C*_ were considered first, the vaccination of the 20-64 years old should be considered first, and the 5-19 years old should be considered second.

**Table 5:** The final outcomes with the optimal age-specific distributions vs. the uniform distribution and no vaccinating for the three countries: China, Brazil and Indonesia.

China Optimal Distribution	No vaccinating	Uniform distribution Beta(1,1)	Min (*R*_0_) Beta(20,12)	Min (*I*_*C*_) Beta(14,20)	Min (*D*_*C*_) Beta(13,20)
*R*_0_	3.737	3.5386	3.47	3.543	3.546
Cumulative infection	14397	103880	100360	93900	94045
Cumulative death	32197	16930	16410	15561	15541
Brazil Optimal Distribution	No vaccinating	Uniform distribution Beta(1,1)	Min (*R*_0_) Beta(20,11)	Min (*I*_*C*_) Beta(20,12)	Min (*D*_*C*_) Beta(20,6)
*R*_0_	3.254	3.008	2.9424	2.9442	2.9555
Cumulative infection	1467001	1505561	768821	764826	1044222
Cumulative death	266300	231402	149687	156072	116915
Indonesia Optimal Distribution	No vaccinating	Uniform distribution Beta(1,1)	Min (*R*_0_) Beta(20,12)	Min (*I*_*C*_) Beta(20,12)	Min (*D*_*C*_) Beta(20,3)
*R*_0_	3.121	2.9187	2.7831	2.7831	2.9647
Cumulative infection	5644001	3962601	2872691	2872691	4543218
Cumulative death	874500	745061	813181	813181	532543

**Fig 4:**
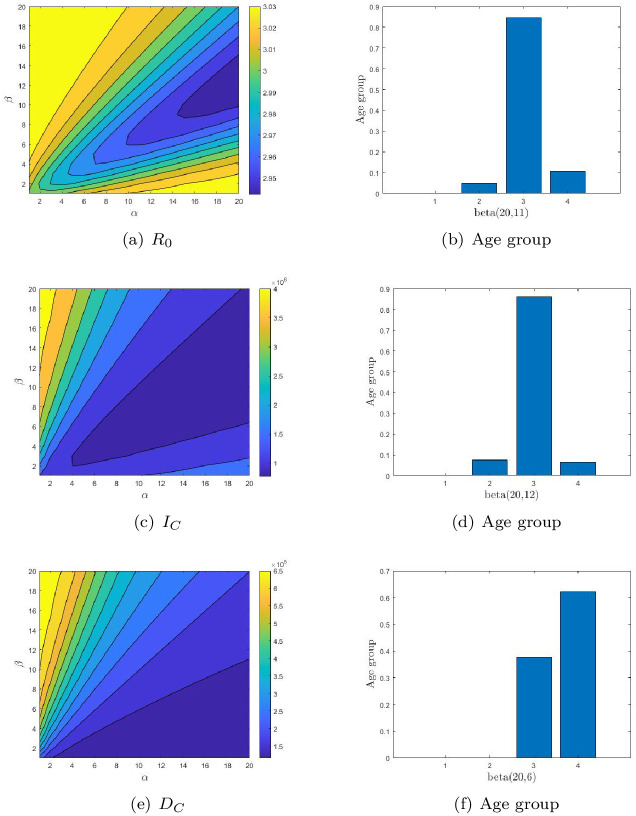
The contour plot of the three endpoints: The basic reproduction number (*R*_0_, 1st row), the cumulative number of infections (*I*_*C*_, 2nd row) and the cumulative number of deaths (*D*_*C*_, 3rd row) for Brazil. The optimal age-specific vaccination distributions for these three endpoints are shown in (b), (d) and (f) respectively.

For Brazil, *R*_0_ is very different from *I*_*C*_ and *D*_*C*_ (See Fig 4). To minimize *I*_*C*_ and *D*_*C*_, OAVD should be the same, and 5-19 should be considered first, then 20-64. When *R*_0_ is minimized, vaccination is preferred between 5 and 19 years of age. In general, in order to control *D*_*C*_, priority should be given to inoculating the elderly, because the mortality rate of the elderly is much higher. Prioritizing young people can also keep *D*_*C*_ below 130, 000, while also ensuring that *R*_0_ grows slowly (Table 5). It was also noted that priority child vaccination was ineffective in controlling the COVID-19 epidemics. Compared with unified vaccination strategy, the optimal age-specific vaccination strategy can reduce *I*_*C*_ or *D*_*C*_.

**Fig 5:**
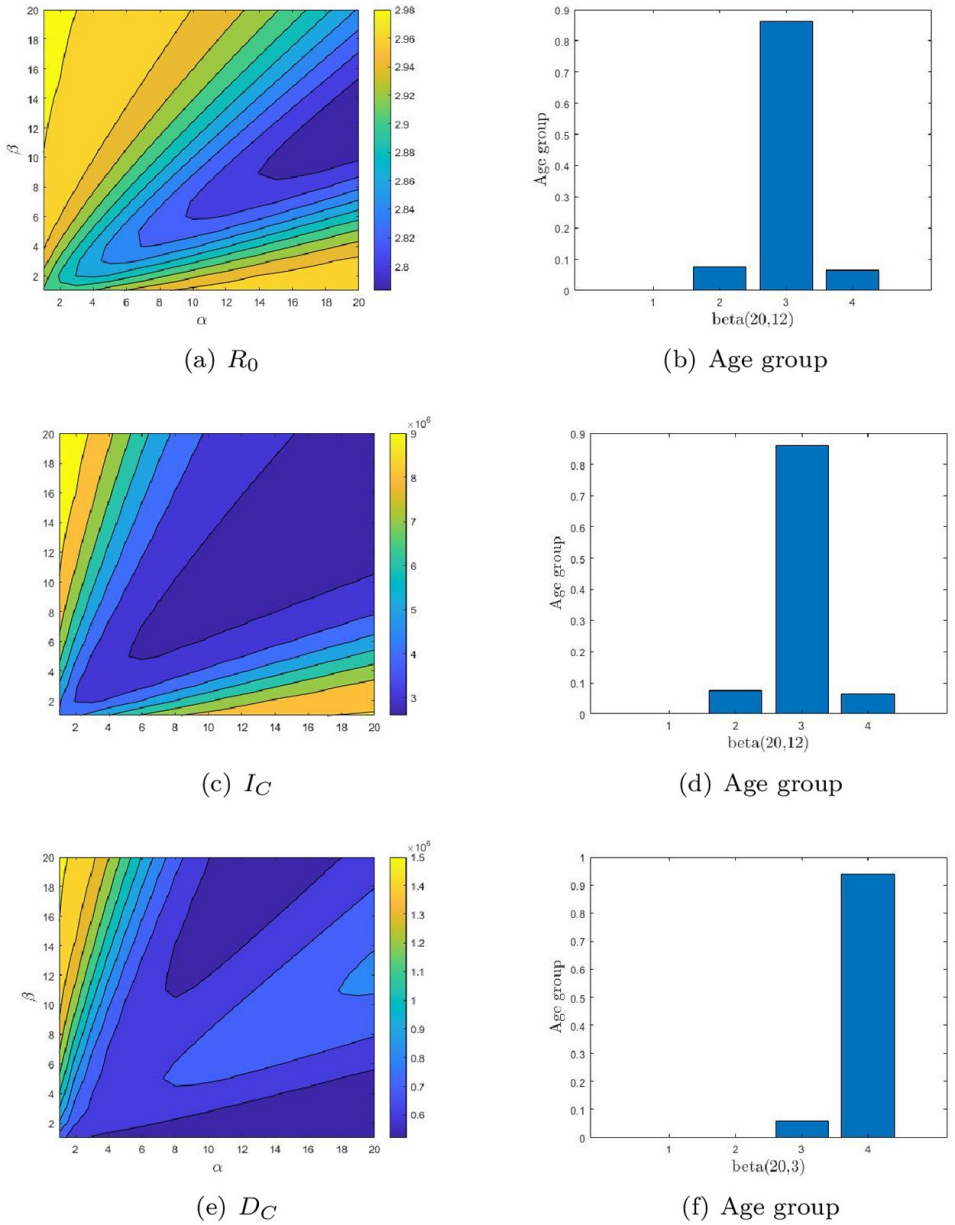
The contour plot of the three endpoints: The basic reproduction number (*R*_0_, 1st row), the cumulative number of infections (*I*_*C*_, 2nd row) and the cumulative number of deaths (*D*_*C*_, 3rd row) for Indonesia. The optimal age-specific vaccination distributions for these three endpoints are shown in (b), (d) and (f) respectively.

For Indonesia, the effects of age-structured inoculation on the three endpoints were similar to those in Brazil, both of OAVDs are similar. Therefore, in order to control *I*_*C*_, it should first vaccinate the young and middle-aged. If the most important is to control *D*_*C*_, it should more vaccinate to the elderly (See Fig 5). In China, the effect of the distribution of inoculation age structure on *R*_0_ and *I*_*C*_ is similar to that in Brazil and Indonesia. However, the influence of age distribution of inoculation on *D*_*C*_ varies greatly, and the OAVD obtained by minimizing *D*_*C*_ is different to that of the other two endpoints.

### 5.2 The vaccination priorities of age structure

In order to directly find out which age group has the best prevention and control effect, we have evaluated the effect of different vaccination sequence in each age group. Since priority vaccination of children was not effective in controlling the COVID-19 epidemic, we compare three indices of control strategies which are random vaccination for six different vaccination sequences. We choose the vaccine administration policy which is assigning to one group after another to carry out. The three indices are *R*_0_, *I*_*C*_ and *D*_*C*_.

For China, the trend of *I*_*C*_ and *R*_0_ is similar. As shown in Table 6, in order to minimize *I*_*C*_ in China, the third group should be vaccinated first. This suggests that for China, the priority of vaccination should be given to young people. Early vaccination of the elderly can effectively reduce *D*_*C*_.

**Table 6:** *R*_0_, *I*_*C*_ and *D*_*C*_ were inoculated in six sequences, China.

The sequence of vaccination	*R*_0_	*I*_*C*_	*D*_*C*_
2-3-4-1	3.437	1.15 × 10^5^	2.71 × 10^4^
2-4-3-1	3.437	1.16 × 10^5^	2.69 × 10^4^
3-2-4-1	3.58	1.15 × 10^5^	2.66 × 10^4^
3-4-2-1	3.58	1.139 × 10^5^	2.66 × 10^4^
4-2-3-1	3.61	1.175 × 10^5^	2.65 × 10^4^
4-3-2-1	3.61	1.17 × 10^5^	2.68 × 10^4^

For Brazil, giving priority to young people led to fewer infections, while giving priority to older people led to fewer deaths(See Table 7). This suggests that in Brazil, priority should be given to vaccinating adolescents and young people, to reduce *R*_0_ and *I*_*C*_. This may be because *I*_*C*_ is directly associated with transmission of infection in the SEIR model.

**Table 7:** *R*_0_, *I*_*C*_ and *D*_*C*_ were inoculated in six sequences, Brazil.

The sequence of vaccination	*R*_0_	*I*_*C*_	*D*_*C*_
2-3-4-1	3.24	1.6 × 10^6^	2.26 × 10^5^
2-4-3-1	3.242	1.87 × 10^6^	2.35 × 10^5^
3-2-4-1	3.47	1.95 × 10^6^	2.44 × 10^5^
3-4-2-1	3.478	2.199 × 10^6^	2.72 × 10^5^
4-2-3-1	3.49	1.76 × 10^6^	2.21 × 10^5^
4-3-2-1	3.492	2.55 × 10^6^	2.38 × 10^5^

In Indonesia, the priority age for vaccination is different from Brazil. Therefore, the control of *I*_*C*_ and *D*_*C*_ should be given priority to the 2nd group (See Table 8). The more 2nd group vaccinated, the better the control. The trend of *D*_*C*_ is similar with China, the earlier the elderly were vaccinated, the lower the death rate.

**Table 8:** *R*_0_, *I*_*C*_and *D*_*C*_ were inoculated in six sequences, Indonesia.

The sequence of vaccination	*R*_0_	*I*_*C*_	*D*_*C*_
2-3-4-1	2.871	3.75 × 10^6^	9.91 × 10^5^
2-4-3-1	2.873	3.55 × 10^6^	1.02 × 10^6^
3-2-4-1	3.03	4.01 × 10^6^	9.3 × 10^5^
3-4-2-1	3.04	3.89 × 10^6^	9.72 × 10^5^
4-2-3-1	3.2	4.81 × 10^6^	8.95 × 10^5^
4-3-2-1	3.21	4.71 × 10^6^	8.882 × 10^5^

## 6 Discussions and conclusions

The COVID-19 is still an important issue that urgently needs to be resolved in the world, and many people lose their lives every day. With the development and use of the vaccine, there is a significant opportunity to end the COVID-19 pandemic, but effective vaccination strategies are essential if we want to quickly control the spread of the pandemic and return to pre-pandemic policies by the vaccine. However, due to the limited production capacity of the vaccine at the present stage, the storage and transportation capacity of the vaccine is insufficient, which cannot guarantee all the people who are willing to be vaccinated. According to the characteristics of different populations, it is particularly important to develop a sequential vaccination strategy. With limited vaccine resources, we should try our best to minimize the number of people affected and the damage caused by the epidemic. Therefore, people with underlying diseases or high risk should be vaccinated first [43]. However, how to allocate vaccine resources rationally after ensuring the vaccination of high-risk groups? In this study, we use an SEIR modelling to investigate the optimal age-specific vaccination strategy. In contrast to the previous literature, we use a continuous function, Beta distribution, to approximate the age-specific vaccination distribution, so we can conveniently optimize the age distribution by minimizing Beta distribution parameters (*α*, *β*) at the three endpoints.

Our results suggest that OAVD and vaccination priorities are different for different outcomes. To minimize *D*_*C*_, vaccination should be given priority to the elderly, while vaccination should be given priority to young people in order to minimize *I*_*C*_. This general principle can vary from country to country due to the age structure of the population. For example, in China, preferential inoculation of middle-aged people can reduce *I*_*C*_ and *D*_*C*_, which may be due to the complex interaction between the age structure of the population and strong government controls. Since the results for *R*_0_ and *I*_*C*_ are similar, presumably because *R*_0_ is directly related to the spread of infection.

With the availability of an effective vaccine, we can expect a rapid return to normal life and regular social contact. However, in the vaccination stage, if the vaccination strategy most suitable for the country is not formulated, it is easy to result a lot of medical resources are wasted. The epidemic is not effectively controlled, which may lead to large-scale *I*_*C*_ and *D*_*C*_, resulting in the waste of vaccine resources. A good strategy is to focus on breakthroughs and think step by step.

In conclusion, our results suggest that the age structure of the population has a significant impact on the effectiveness of age-specific vaccination strategies, and that the vaccination sequence should vary with age outcomes. In the context of limited vaccine resources, different age-priority guidelines for the general population need to be considered in order to control the COVID-19 pandemic more effectively. In addition, different countries need to develop specific vaccination strategies based on the age structure of their populations. The SEIR model of age structure also analyzed the priority sequence of vaccination in different age groups, and compared with simultaneous vaccination, better effects could be achieved for specific populations. In order to get OAVD, we minimize the three endpoints. Although it may fall on the boundary of the parameter (*α*, *β*) search space, it does not affect the priority of the inoculated population. Vaccination strategies need to be further integrated with the effectiveness of the vaccine and the effectiveness of other control measures. Novel coronavirus continues to mutate, with South African and Brazilian variants lowering the protection rates of some vaccines. The future direction of COVID-19 vaccine development is likely to be similar to the influenza vaccine strategy, which uses a polyvalent vaccine against the virus.
